# Application of Diversity Combining with RLS Adaptive Filtering in Data Transmission in a Hydroacoustic Channel

**DOI:** 10.3390/s20247255

**Published:** 2020-12-17

**Authors:** Agnieszka Czapiewska, Andrzej Luksza, Ryszard Studanski, Andrzej Zak

**Affiliations:** 1 Faculty of Electronics, Telecommunications and Informatics, Gdansk University of Technology, Narutowicza 11/12, 80-233 Gdańsk, Poland; agnieszka.czapiewska@pg.edu.pl; 2 Faculty of Electrical Engineering, Gdynia Maritime University, Morska 81-87, 81-255 Gdynia, Poland; a.luksza@we.umg.edu.pl; 3 Faculty of Mechanical and Electrical Engineering, Polish Naval Academy, Smidowicz 69, 81-127 Gdynia, Poland; a.zak@amw.gdynia.pl

**Keywords:** hydroacoustic channel, diversity combining, RLS adaptive filter, data transmission

## Abstract

When transmitting data in a hydroacoustic channel under difficult propagation conditions, one of the problems is intersymbol interference (ISI) caused mainly by the effect of multipath propagation. This phenomenon leads to a decrease in transmission parameters, and sometimes completely prevents it. Therefore, we have made an attempt to use diversity combining with Recursive Least Squares (RLS) adaptive filtering to improve the quality of data transmission in a hydroacoustic channel with strong reflections. The method was tested in simulation and during measurements in a real environment. The influence of the method on data transmission in the hydroacoustic channel was examined in detail. The obtained results allow us to draw conclusions regarding the purposefulness of use of diversity combining and RLS adaptive filtering in order to improve the quality of data transmission by reducing the effect of ISI.

## 1. Introduction

Sound travels through water better than the other forms of radiation. Thus, underwater sound has many applications in the areas of human activity at sea. The applications of the underwater acoustic wave have their origin deep in the past—before the World War I, and later—when a huge progress was made in instruments and devices. They become useful during underwater works, marine environment research, monitoring fish, plankton and aquatic vegetation and underwater sports and are particularly important from the military point of view. Due to the development of quickly deployed underwater observation systems as well as underwater robotics, in particular autonomous underwater vehicles, a need for wireless data transmission has arisen. At the moment, the hydroacoustic channel remains the best transmission medium [1]. Unfortunately, despite great progress in the field of telecommunications, the possibility of utilization of an underwater acoustic channel (UAC) for communication is very limited and there is still intensive research on new solutions [2,3]. Channel limitations result from complex physical phenomena related to sound propagation in water. One of the most important phenomena here is the multipath propagation, which occurs especially in the case of shallow waters, narrow reservoirs or reservoirs with intensive hydrotechnical buildings (canals, ports, etc.), as well as in the case of a strong stratification of water caused by the temperature or salinity, which can be observed in the basin of the Baltic Sea. It causes many reflections which lengthen the hydroacoustic channel memory and leads to inter-symbol interference including tens or hundreds of symbols, which makes communication much more difficult [4,5,6].

This research focuses on the assessment of the impact of diversity combining reception with Recursive Least Squares (RLS) adaptive filtration on the quality of data transmission in a hydroacoustic channel in difficult propagation conditions.

Underwater communication methods are widely discussed in the literature [1,7,8,9]. Usually, attempts are made to apply methods known and proven in radio communication, especially in mobile telephony. The problem of data transmission in a hydroacoustic channel in difficult propagation conditions is also quite often described and discussed in the literature [6,10,11,12]. Various methods are sought to counteract such phenomena as intersymbol interference [3,13,14]. However, the use of diversity combining in underwater communication is rarely mentioned [15,16,17,18,19] despite the fact that the methods of diversity combining are quite well described and their applications, especially in radio communication, are often presented [20,21,22]. Adaptive filtration methods and their usefulness in channel equalization [23,24,25] have also been known for a long time. Various methods of determining adaptive filters’ coefficients are presented in the literature. The most popular are the Least Mean Squares and Recursive Least Squares algorithms [26,27,28,29]. There are also reports of the use of neural networks for channel correction [30,31,32]. Despite the fact that individual processing methods are described in the literature, the authors of this article have not come across a solution that simultaneously applies diversity combining and correction with the use of adaptive filters. Neither have the authors come across comprehensive research results that could explain in what circumstances the use of adaptive filtration methods would improve the received signal quality. Therefore, it was decided to investigate the effect of the combined use of diversity combining and adaptive filtering on the quality of data reception in the hydroacoustic channel. In general, the use of adaptive filtering is limited in the case of a low signal-to-noise ratio (SNR), so it was decided to conduct detailed studies that would indicate under which conditions the applicability of this solution is justified. It is also known that diversity combining improves the SNR as well as reducing the inter-symbol interference by decreasing the amplitude of replicas of transmitted signal. In addition to that, it is expected that in the case of using diversity combining it will be possible to extend the scope of application of adaptive filtration. The proposed solution of the combined use of these two techniques should result in an increase in reception quality.

The article is organized as follows: Section 2 describes in detail the methods used during the research as well as the adopted assessments’ indicators; Section 3 presents and discusses the obtained results of simulation tests as well as the results obtained in real conditions, including the impact of the proposed method on the quality of data transmission in the underwater acoustic channel; Section 4 presents the conclusions resulting from the conducted research and indicates further research directions.

## 2. Methods Description

### 2.1. Diversity Combining

We assume that the transmitting–receiving circuit has a classic configuration, i.e., on the transmitting side there is one hydrophone generating an acoustic wave and on the receiving side there are several (two or more) receiving hydrophones.

It was assumed that the transmitted signal $x\left(n\right)$ is a sine wave (with the frequency $f_{c}$) modulated by a random sequence $d\left(n\right)$, which can be written as:

(1) $$x\left(n\right)=d\left(n\right)\sin\left(2\pi f_{c}n\right)$$

where: $d\left(n\right)$—data sequence, $f_{c}$—carrier frequency, n—number of sample.

The transmitted signal is filtered using a raised cosine (RRC) filter with the roll-off factor equal to 0.4. This is to minimize the spectral impact beyond the adopted band, which will ensure compatibility with other systems and devices operating in this propagation environment.

The receiving hydrophones are positioned at a specific and fixed positions in relation to each other, forming the receiving antenna. The hydrophones in the antenna receive the signal under slightly different conditions, i.e., convoluted with a different channel impulse response. This response will characterize the propagation conditions of an acoustic wave on the way from the transmitter to a specific receiver, taking into account the multipath propagation of the signal. Therefore, it can be expressed as follows:

(2) $$y_{k}\left(n\right)=\mathrm{conv}\left(x\left(n\right),g_{k}\left(n\right)\right)+s_{k}\left(n\right)$$

where: $y_{k}\left(n\right)$—signal received in k-th channel, $x\left(n\right)$—transmitted signal, $g_{k}\left(n\right)$—channel impulse response characteristic for k-th channel, $s_{k}\left(n\right)$—noise (random process).

In the simplest solution, to perform diversity combining, the signals from all the channels should be summed with the same weights (so-called even summation). Since the signal propagation path for each hydrophone may be different, due to the position of the transmitter in relation to the receiver, a coherent reception for the first arriving signal must be ensured. Therefore, the diversity combining process based on even summation can be described by the equation [20]:

(3) $$u\left(n\right)=u_{1}\left(n+n_{1}\right)+u_{2}\left(n+n_{2}\right)+\ldots +u_{k}\left(n+n_{k}\right)$$

where: $u\left(n\right)$—signal after diversity combining, $u_{k}\left(n\right)$—signal received in k-th channel, $n_{k}$—signal delay in k-th channel expressed in samples.

**Figure 1:**
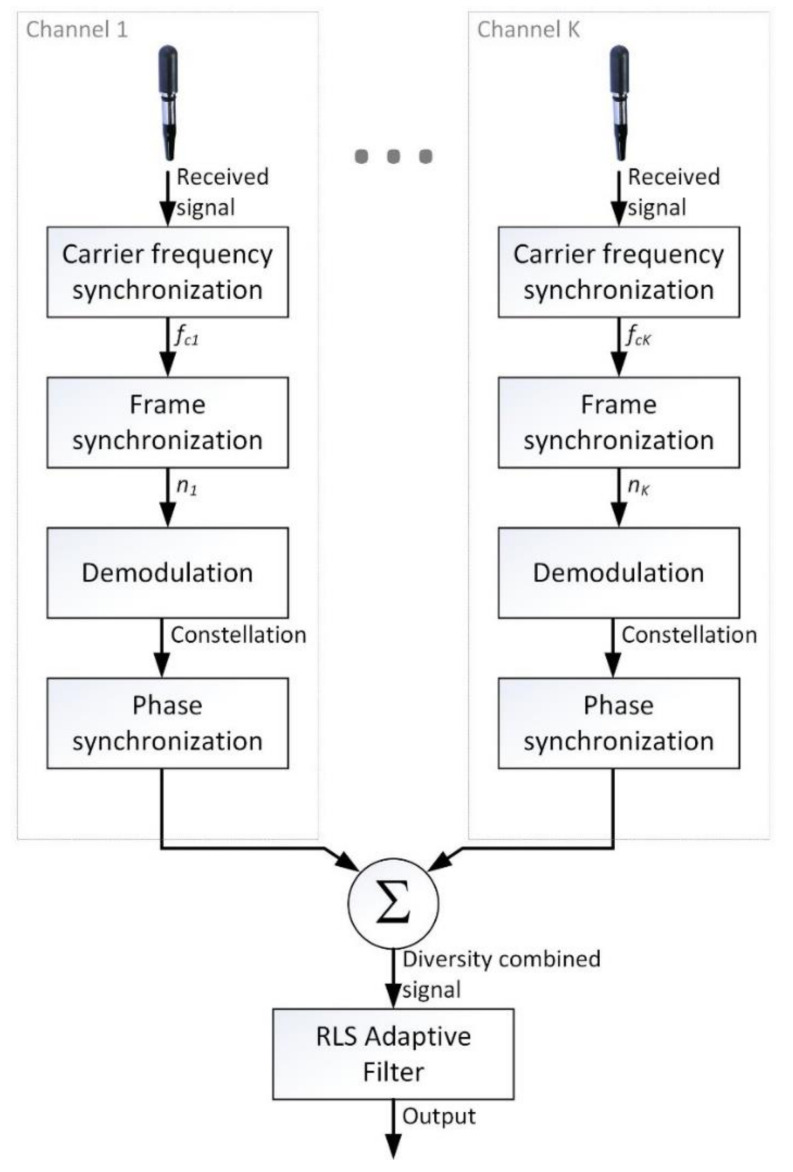
Block diagram of the receiver.

The signal processing diagram in the receiver is shown in Figure 1.

The input signal is the voltage at the output of the k-th hydrophone. First, synchronization of the carrier frequency of the received signal is performed. Then, frame synchronization is performed using the known preamble transmitted at the beginning of each frame. During the frame synchronization, the received signal is correlated with a reference signal (modulated preamble) and the detection of the correlation maximum is performed. The found maximum indicates the start of the frame. This is followed by demodulation and phase correction. The signals from each receiving channel prepared this way are added algebraically. Subsequently, the collective signal is processed using the RLS adaptive filter.

### 2.2. RLS Adaptive Filter

In order to reduce the multipath effect in the receiving path, an adaptive filter, with the Recursive Least Squares (RLS) algorithm determining its coefficients, was used. This choice was primarily dictated by the fact that the selected adaptive filter counteracts strong intersymbol interference and is characterized by high resistance to the Doppler effect [33]. 

**Figure 2:**
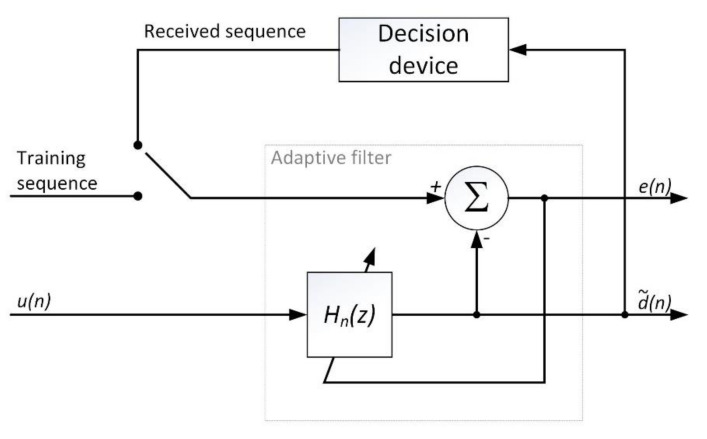
Adaptive channel correction system.

Adaptive channel correction can be performed in a circuit, where the input signal is the received sequence $u\left(n\right)$, and the reference signal is a known training sequence. The task of the filter is to convert the received sequence $u\left(n\right)$ into the sequence closest to the one sent $d\left(n\right)$. Figure 2 shows the system of adaptive channel correction, where $\tilde{d}\left[n\right]$—estimate of the transmitted sequence, $e\left(n\right)$—adaptation error, $H_{n}\left(z\right)$—transfer function of the correction filter. The system assumes that an Infinite Impulse Response (IIR) filter will be used. The number of filter taps depends on the maximum channel delay. The length of the training sequence must be two times longer than the maximum channel delay.

### 2.3. Evaluation of Transmision Quality

To determine the Bit Error Rate (BER) value, a large amount of data should be transmitted; it is assumed that 100 times more bits should be sent than the absolute value of the order of the expected BER value (e.g., if we expect a BER of 10^−3^, then 100,000 bits should be sent). During the measurements in real conditions, the required number of transmissions is difficult to obtain. Since we use Binary Phase Shift Keying (BPSK) modulation, only the inphase values have an impact on the decisions about the transmitted bits. Therefore, to determine the reception quality, a parameter was used that was determined on the basis of the constellation spread obtained for a given frame for the inphase value. We will refer to this parameter as $I_{quality}$ hereafter.

To calculate this parameter, we will use the following formula:

(4) $$I_{quality}=\frac{\mu_{u_{I}}}{\sigma_{u_{I}}^{2}}$$

where: $\mu_{u_{I}}$—average of inphase values, $\sigma_{u_{I}}^{2}$—variance of inphase values. 

In order to map the value of the aforementioned parameter to the BER values, simulations with the Monte Carlo method were performed. During the simulations, transmissions were made for different Signal-to-Noise Ratio (SNR) values so as to obtain reliable information about the BER value. The following transmission parameters were adopted:

− Sampling frequency: 500 kHz;
− Carrier frequency: 100 kHz;
− Signal bandwidth: 100 kHz;
− BPSK modulation.

**Figure 3:**
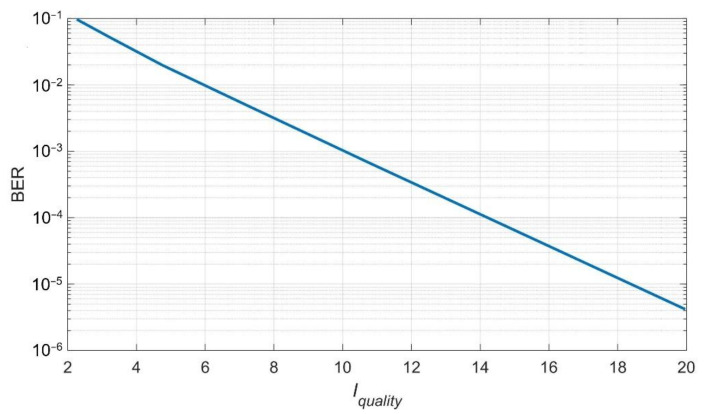
The relation between the BER and the $I_{quality}$.

For each transmission, the BER and the $I_{quality}$ parameter were determined and for a given SNR value they were averaged over all the implementations. Figure 3 shows the experimentally obtained (in a simulation environment) relation between the transmission quality (expressed by the BER coefficient) and the proposed $I_{quality}$ parameter.

The obtained results show that the $I_{quality}$ parameter value can be clearly mapped to the transmission quality expressed by the BER.

## 3. Research Results

The research was conducted in two stages. In the first stage, simulation tests with the Monte Carlo method were carried out, which allowed verification of the adopted solutions and the drawing of preliminary conclusions regarding the suitability of the methods used. In the second stage, tests were carried out under real conditions in a lake. These tests enabled the verification of the results obtained in the simulation stage.

### 3.1. Simulation Experiment

Simulation experiments were carried out with the Matlab environment using the Monte Carlo method. The simulation parameters were selected taking into account the possibility of conducting the research in real conditions. The following simulation parameters were adopted:

− BPSK modulated signal;
− Number of bits transmitted in the frame: 1260;
− Preamble length (pilot): 126 bits;
− Modulated signal bandwidth: 100 kHz;
− Modulation rate: 50 kBd;
− Sampling frequency: 500 kHz;
− Carrier frequency: 100 kHz;
− Number of replicas: 1 ÷ 15;
− Amplitude of the replicas: uniformly distributed random numbers;
− Delay of replicas: uniformly distributed random numbers from 0 to 1 ms.

The amplitudes of the replicas were normalized in such a way that their total algebraic power was equal to an assumed value. For this purpose, the SRR (Signal-to-Replicas Ratio) parameter was introduced, expressing the ratio of the signal’s first replica power to the total replica power, which can be written:

(5) $$SRR_{lin}=\frac{P_{1}}{\sum_{r=2}^{R+1}P_{r}}$$

where: $P_{1}$—signal’s first replica power, $P_{r}$—power of r-th replica, R—number of replicas.

(6) $$SRR_{dB}=10\;log\frac{P_{1}}{\sum_{r=2}^{R+1}P_{r}}$$

Diversity combining was performed coherently for the signal’s first replica in the baseband. In the tests, the number of training bits was assumed to be 100, which includes twice the maximum delay time of the replica (twice the time of the maximum delay (assumed to be 1 ms) multiplied by the modulation rate (assumed to be 50 kBd)). The results obtained within one characterization come from two million transmitted frames.

During the simulation of the diversity combining, two approaches to generating an impulse response were adopted. In the first approach, the impulse response was randomized for each of the receivers. In this case, the amplitude and the delay were random numbers. Amplitude was randomized in the range from −1 to 1, and the delay in the range from 0 to 1 ms for each replica. The final form of the impulse response depends on the number of replicas adopted as well as their amplitudes and delays. In the other approach, the impulse response was randomized for the first receiver, and for the remaining ones, the changes of individual replicas over time in the range of $\left[-\Delta t,\;\Delta t\right]$ and the amplitude in the range of $\left[-\Delta A,\;\Delta A\right]$ were randomized according to the uniform distribution.

#### 3.1.1. Noise Characteristics

**Figure 4:**
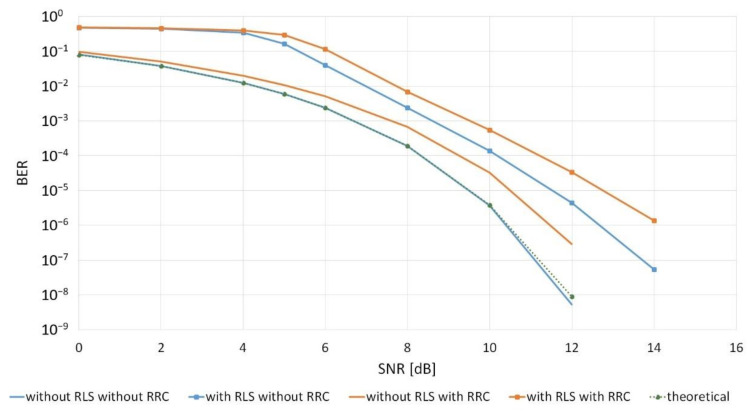
Bit error rate for the BPSK reception with and without RLS adaptive filtering for a single receiver.

In the first stage, the influence of the RLS adaptive filter on the reception quality expressed in BER as a function of the signal power to the noise power ratio SNR was investigated. The quality of a traditional BPSK signal reception was compared with the reception, in which RLS adaptive filtering was applied, both with and without the RRC filter in the transmitting path. The results of the simulation experiment are shown in Figure 4. 

Figure 4 shows that the theoretical curve coincides with the curve obtained from the simulation without the use of the RLS and RRC filter, which proves that the simulation has been correctly implemented. Moreover, the prediction that the RLS filter in the presence of noise did not improve the reception quality was confirmed. The use of the RRC filter, due to the fact that it interferes with the spectrum of the transmitted signal, deteriorates the reception quality.

In addition, the simulator reception quality was compared for the traditional reception (without the use of the RRC raised cosine filter and without the RLS adaptive filter) with the analytical dependence expressed as follows (Figure 4) [34]:

(7) $$BER=\frac{1}{2}\mathrm{erfc}\left(\sqrt{\frac{E_{B}}{N_{0}}}\right)$$

where: $E_{B}$—bit energy, $N_{0}$—noise power spectral density.

The obtained results prove the correct implementation of the simulator.

For the purposes of further research, it was assumed that an RRC filter would always be used in signal formation.

**Figure 5:**
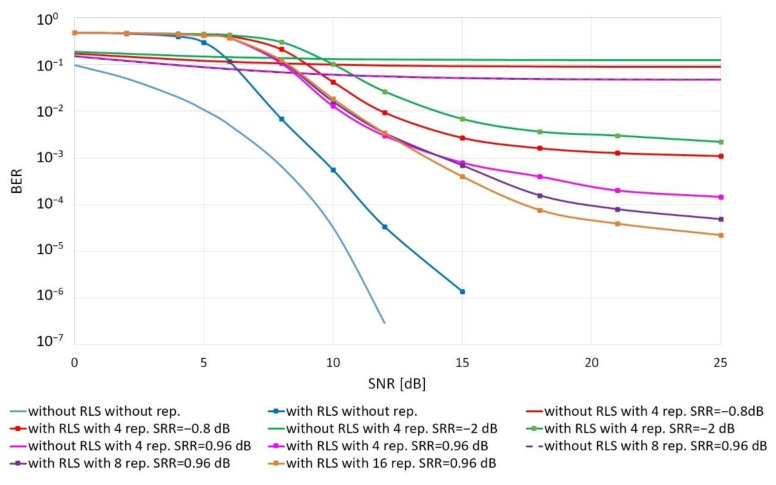
Quality of data transmission in the replica channel as a function of the signal power to the noise power ratio for a single receiver.

Figure 5 shows the quality of the reception as a function of the signal power to the noise power ratio for a different number of replicas and the $SRR_{dB}$ value.

The obtained results show that if the noise is the only interference, the higher quality is ensured by a coherent way of receiving without applying the RLS adaptive filter. Adaptive filtration requires a high SNR. If the SNR is less than 5 dB, the reception using the adaptive filter is not possible. The exact SNR value for which it makes sense to use an adaptive filter also depends on the SRR parameter. The higher it is, the lower the SNR values; the use of the RLS filter improves the reception quality.

#### 3.1.2. Tests of the Reception Quality Dependence on the Number of Replicas

**Figure 6:**
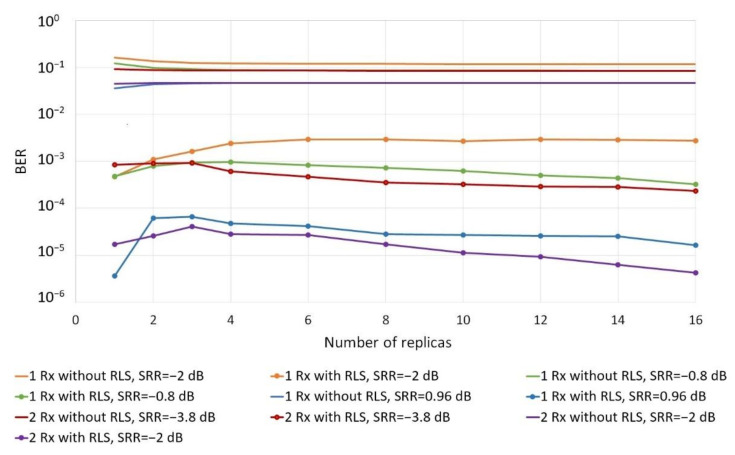
Comparison of the reception quality as a function of the number of replicas at SNR = 30 dB.

In the next stage, the influence of the number of replicas on the reception quality was examined with the use of one and two receivers. The results are shown in Figure 6.

As a result of the research, it can be concluded that even with a large number of replicas and their relatively high total power (higher and comparable with the power of the signal’s first replica), RLS adaptive filtering ensures at least a tenfold improvement in quality. With the increase in the number of replicas above four, the reception quality does not deteriorate. It was also noted that the reception quality without the RLS adaptive filter, if the number of replicas is at least two, does not depend on their number, but mainly on their power.

It should be noted that if the replicas do not add up and only the signal’s first replica is subject to the coherent addition of signals from individual receivers during the collective reception, the value of the SRR parameter for diversity combining ($SRR_{lin}^{dc}$) will be as many times greater as the number of the receivers used to create the signal summary. In case when the replicas add up, this relationship would not be so beneficial. Rewriting the formula for the SRR for the i-th receiver, we get:

(8) $$SRR_{lin}^{i}=\frac{A_{i}{}^{2}}{\sum_{j=1}^{J}A_{ij}{}^{2}}=\frac{A_{i}{}^{2}}{P_{Ri}}$$

where: $A_{i}$—amplitude of the signal’s first replica at the i-th receiver, $A_{ij}$—amplitude of j-th replica in i-th receiver, J—number of replicas, $P_{Ri}$—total power of replicas in the i-th receiver.

In diversity combining, since the individual receivers are mutually synchronized according to the signal’s first replica, the amplitudes of the signals’ first replica add up, while the presence of the other replicas is independent, so we can assume:

(9) $$SRR_{lin}^{dc}=\frac{{\left(\sum_{i=1}^{K}A_{i}\right)}^{2}}{\sum_{i=1}^{K}\left(\sum_{j=1}^{J}A_{ij}{}^{2}\right)}$$

where: K—number of receivers. 

As it was assumed in the simulations that $A_{i}$ and $P_{Ri}$ for each receiving path are the same, the above formula can be written:

(10) $$SRR_{lin}^{dc}=\frac{{\left(K\cdot A_{i}\right)}^{2}}{K\cdot P_{Ri}}$$

Hence:

(11) $$SRR_{lin}^{dc}=K\cdot SRR_{lin}^{i}$$

**Figure 7:**
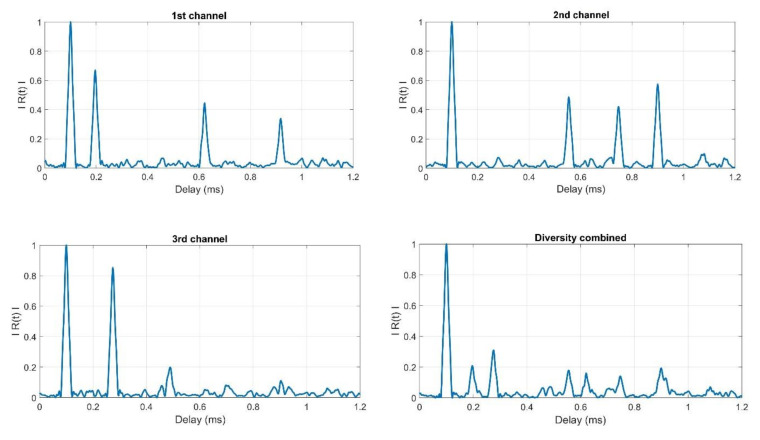
Example showing the impact of diversity combining on the estimate of the hydroacoustic channel impulse response moduli.

The above formulas prove that the value of the SRR parameter increases (improves) as many times as the receiving paths will be used, under the previous assumptions related to the amplitude of the signal’s first replica and the total power of the replicas. This effect can be observed in the estimates of the hydroacoustic channel impulse response modulus (Figure 7). 

As it is shown in Figure 7, after diversity combining, the amplitude of all replicas present in each channel decreased approximately as many times as there are receiving channels. For example, the amplitude of the first replica at the first channel at the time moment 0.2 ms is 0.67. In diversity combined signal, the amplitude of this replica (also present at the time of 0.2 ms) is 0.2, which is almost three times lower because we use three receiving channels. The reduction factor is not exactly equal to the number of receiving channels used. It is caused by the fact that the amplitudes at any given point in time in the remaining channels are non-zero. It should also be noted that all replicas that appear in the individual receiving channels are visible in the signal after the diversity combining.

#### 3.1.3. Tests of the Reception Quality Dependence on the Total Power of the Replicas

**Figure 8:**
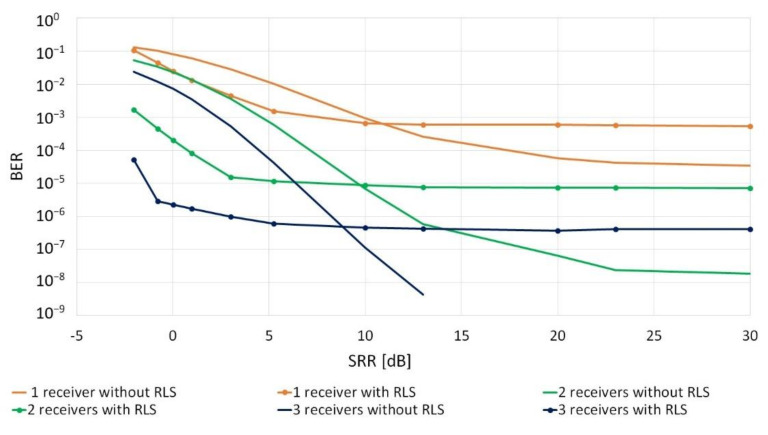
Influence of the total power of the replicas on the reception quality for SNR = 10 dB, the number of replicas = 4.

In the next stage, the influence of the total power of the replicas on the reception quality was examined with the use of one, two and three receivers. An approach was used in which the impulse response was randomized for each of the receiving paths. The results are shown in Figure 8.

The obtained results prove that the use of the adaptive RLS filter is justified only when the value of the total power of the replicas is sufficiently large in relation to the signal’s first replica. The SRR value, at which it is reasonable to use adaptive RLS filtering, depends on the SNR. Then, the quality of the reception at a given SNR coincides with the characteristics from Figure 5.

**Figure 9:**
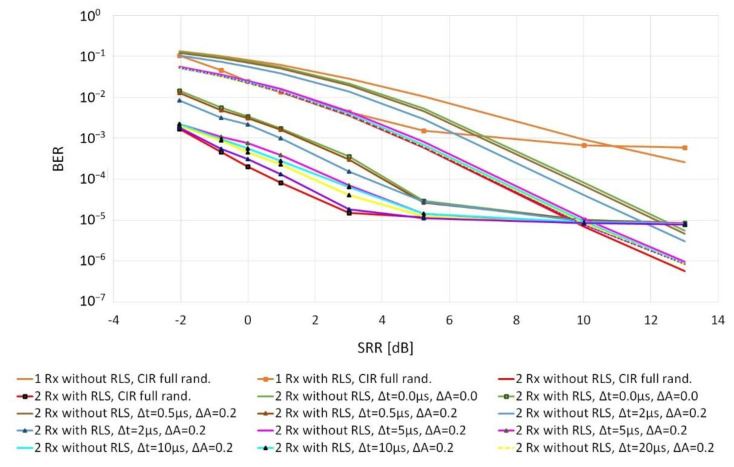
Impact of the differentiation of impulse responses on the quality of diversity combining when using two receivers for SNR = 10 dB and 4 replicas.

Then, the influence of the differentiation of impulse responses between individual receiving paths on the quality of the collective reception was examined. At this stage, the impulse response was randomized for the first receiver, and for the others—its modification was adopted, consisting of shifting the replicas in time by a random value in the range of $\left[-\Delta t,\;\Delta t\right]$ and changing their amplitudes in the range of $\left[-\Delta A,\;\Delta A\right]$. The values of: $\Delta t\;=\;\left\{500\;\mathrm{ns},\;2\;µs,\;5\;µs,\;10\;µs,\;20\;µs,\;50\;µs\right\}$ and ΔA = 0.2 were assumed for the tests. After the modification, it was ensured that the condition of the equal total power of the replicas was met. These simulation test results were compared with the cases, in which the impulse response in individual receivers was the same and fully random (see Figure 9). Figure 9 also shows the reception quality with a single receiver (in orange).

The obtained results indicate that, first of all, the use of the RLS algorithm is justified in the presence of the replicas of the transmitted signal with SRR not greater than 10 dB. Moreover, in the case of diversity combining, it is recommended that the impulse responses in individual reception paths should be as different as possible. If the reception takes place at the same point (the same impulse responses), the replicas will also add up and their power will increase analogously to the power of the signal’s first replica (green color). In this case, the improvement of the reception quality is mainly caused by the reduction in the noise power (noise averaging). Figure 9 shows that with the increase in the Δt parameter, the obtained characteristics reach the values that are more and more similar to the characteristics obtained for independent impulse responses in individual receiving paths (red color). The BER values are consistent with the characteristics shown in Figure 5. For a single receiver, this relationship is visible directly, and for diversity combining with two hydrophones, SNR improves by 3 dB.

### 3.2. Real Conditions Experiment

**Figure 10:**
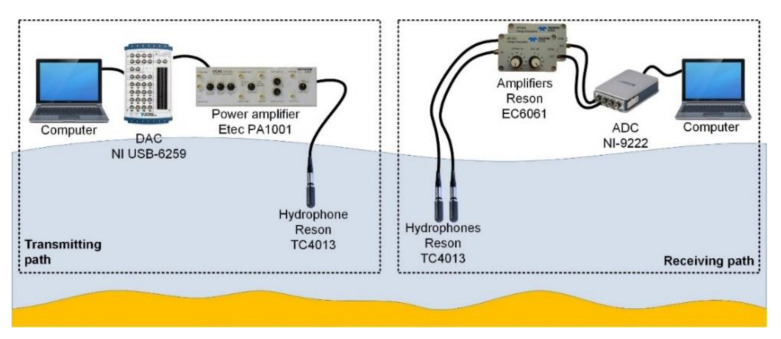
Block diagram of the measurement system.

In order to verify the results obtained during the simulation experiment, tests were carried out in real conditions with the use of physical signals. The research was carried out during the measurement campaign on the Lake Kosobudno (Pomeranian Voivodeship, Poland). The depth of the reservoir at the point of tests was about 3.5 m. The bottom in the experiment area was sandy without any underwater vegetation. During the tests, the water temperature was about 17 °C, the weather was very good, and the wind was weak. The transmitting and receiving hydrophones were lowered to a depth of 2 m from the floating jetty, whose immersion was about 0.4 m, at a distance of about 12 m from the shore. The receiving hydrophones were placed in one line with the transmitting hydrophone. The individual receiving hydrophones were 4 m apart, and the distance between the transmitting hydrophone and the receiving ones was changed in the range from 8 m to 36 m. Figure 10 shows the measuring system, including the transmitting path and the receiving path used during the tests in real conditions.

The transmitting path uses as the projector a TC4013 hydrophone (Teledyne RESON A/S, Denmark) connected to the P1001 power amplifier (Etec A/S, Denmark), which was connected to the NI USB-6259 digital-to-analog converter card (National Instruments, USA), and then to the computer with the NI SignalExpress (National Instruments, USA) software via USB interface. In the receiving path, two TC4013 hydrophones were used, each connected to the EC6061 amplifier (Teledyne RESON A/S, Denmark) and then to the NI-9222 analog-to-digital converter (National Instruments, USA), which was connected via an Ethernet interface to a computer with NI SignalExpress software.

In the real conditions experiment, a sinusoidal signal with BPSK modulation was used. A random sequence was used for modulation. The length of the frame was 1260 bits; the learning sequence length for adaptive filtration was 100 symbols. The carrier frequency of the signal was changed in the range from 40 kHz to 120 kHz. The modulation rate was changed in the range from 5 kBd to 60 kBd. The sampling frequency was set to 500 kHz. All the results presented bellow come from the test where the carrier frequency was 120 kHz and the modulation rate was 15 kBd.

**Figure 11:**
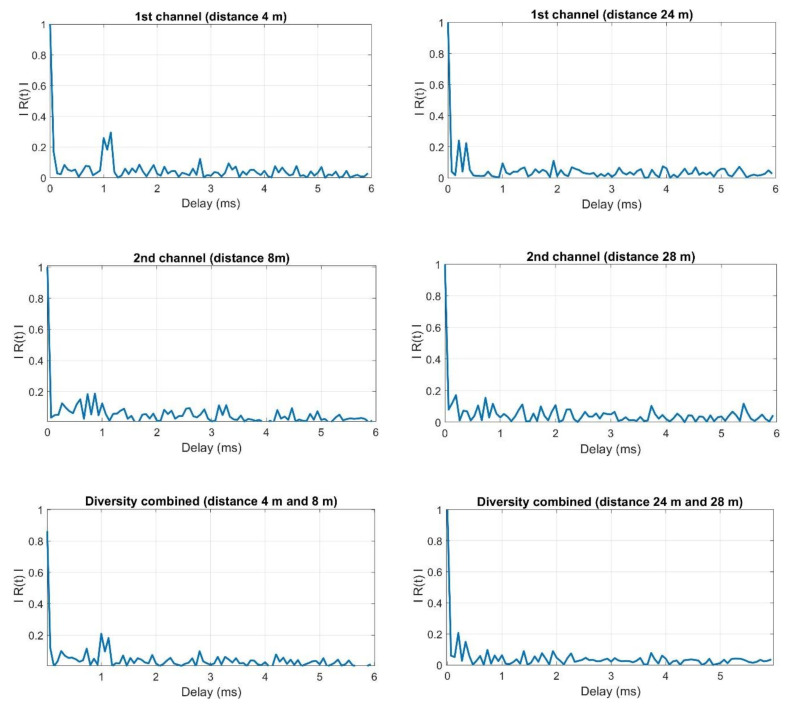
Example of the estimates of the impulse response modules obtained during the tests in real conditions.

Figure 11 shows the estimates of the impulse response moduli determined in accordance with the methodology described in [5] for the reception by individual hydrophones and for diversity combining.

The presented estimates of the impulse response modules show that at the distance of 4 m and 8 m of the receiving hydrophones from the transmitter, the replicas are still clearly visible after diversity combining. In the case of receiving at a distance of 24 m and 28 m, the replicas after diversity combining are on the background level. It should be noted that the correlation background in the case of reception at a distance of 4 m and 8 m will result primarily from the strength of the received signals (the transmitted one and its replicas), while the contribution of noise in the formation of the correlation background is small. In the case of the distance of 24 m and 28 m, the situation is slightly different, namely, the power of the received signals is much smaller, hence the noise determines the strength of the background correlation.

The figures below show the examples of constellations for the reception recorded at the hydrophone–transmitter distances of 4 m and 8 m and 24 m and 28 m, respectively. 

**Figure 12:**
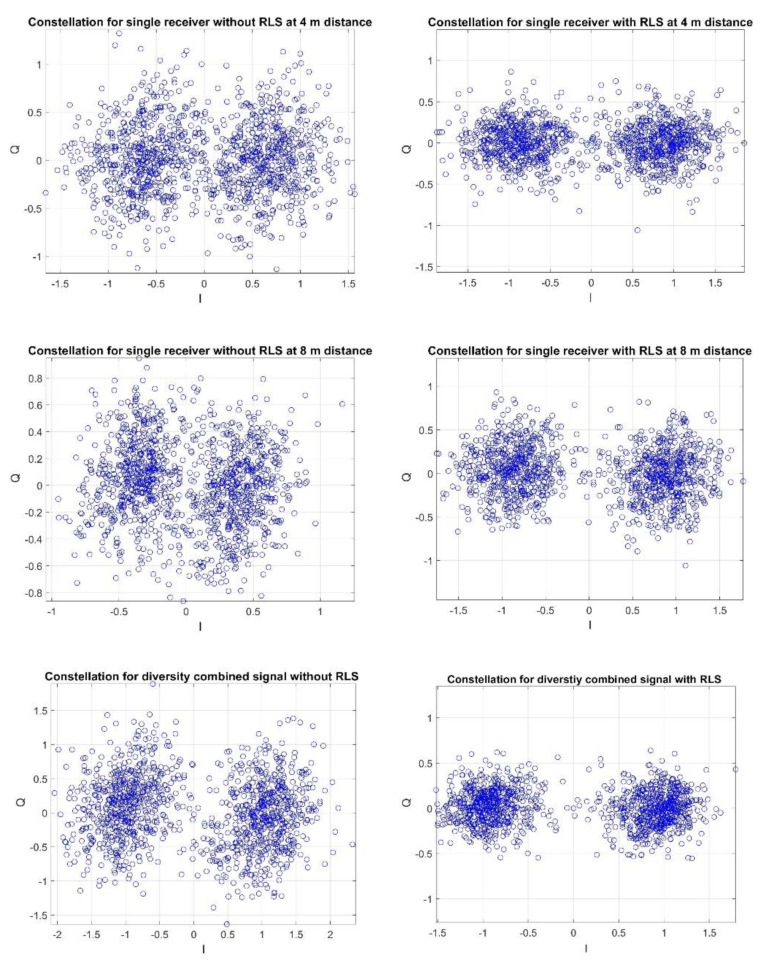
Example of a constellation for reception with hydrophones at the distance of 4 m and 8 m from the transmitter.

**Figure 13:**
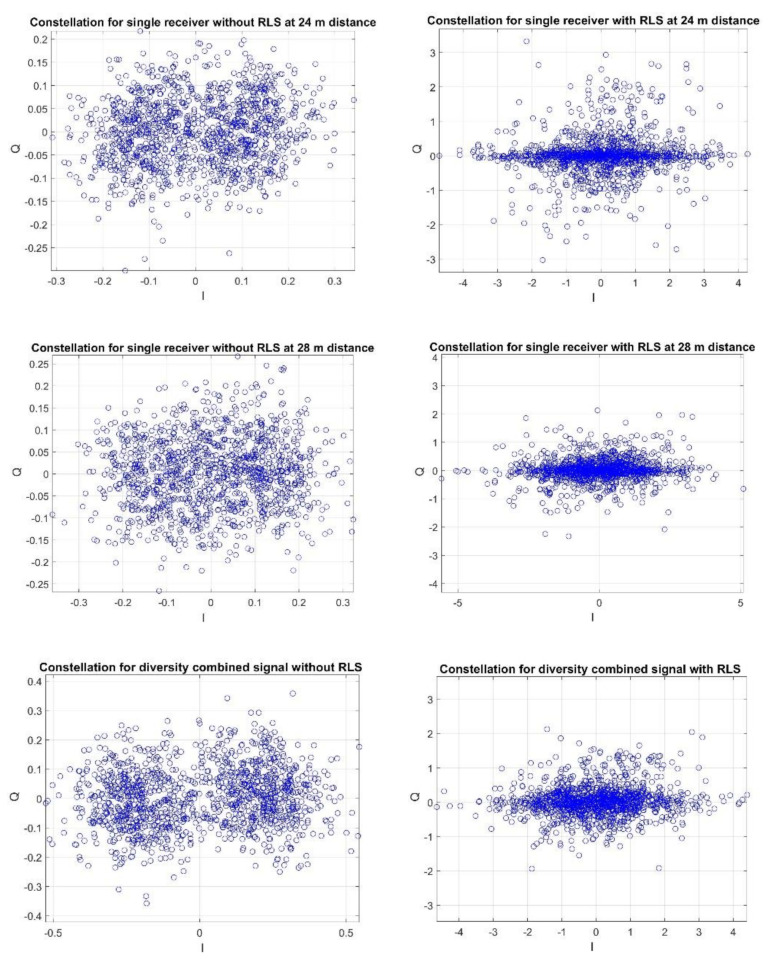
Example of a constellation for reception with hydrophones at the distance of 24 m and 28 m from the transmitter.

Figure 12 clearly shows that the use of the RLS filter for both the single receiver and the diversity combining improves the reception quality, i.e., there is a clear increase in the concentration of the constellation points. In Figure 13, first, we can see a significant deterioration of the signal-to-noise ratio in relation to the case presented in Figure 12. Moreover, the use of RLS filtering does not improve the reception quality. However, it should be noted that diversity combining without RLS improves the reception quality, which can be seen by increasing the concentration of the constellations. This is because diversity combining improves the signal-to-noise ratio.

Table 1 and Table 2 present the exemplary results for the cases, where the receiving hydrophones were located at the distances of: 4 m and 8 m, and 24 m and 28 m from the transmitter, respectively. The values presented in Table 1 and 2 are related with results presented in Figure 12 and Figure 13. The values of $I_{quality}$ prove that for the distances 4 and 8 m the reception quality is greater than for the distances 24 m and 28 m, as the $I_{quality}$ parameter is two times greater for 4 m and 8 m, than for 24 m and 28 m. Additionally, the reception quality improvement is clearly visible for the closer distances after the RLS utilization, as the $I_{quality}$ parameter doubles. The results in Table 2 coincide with the conclusions presented for Figure 13. The received SNR is smaller than for the closer distances, which is proven by the low values of $I_{quality}$ parameter and the utilization of RLS adaptive filter does not improve the reception quality. However, after diversity combining the value of the $I_{quality}$ parameter increases.

The obtained results indicate that at the distances of 4 m and 8 m, the use of the RLS filter is justified as there are clearly relatively high power replicas. The quality with the RLS is clearly better not only through BER, but also through the $I_{quality}$ parameter in relation to the reception without the RLS. In the case of the reception at the distance of 24 m and 28 m, the replicas are on the background level, therefore the use of the RLS leads to a deterioration in quality, while diversity combining is justified. It should be noted that these conclusions are consistent with those resulting from the simulation tests.

## 4. Conclusions

One of the main problems in the data transmission in a hydroacoustic channel in difficult propagation conditions is the occurrence of inter-symbol interference. It negatively affects the quality of the transmission and at the same time can cause a loss of communication.

The main purpose of the study was the assessment of the impact of diversity combining reception with Recursive Least Squares adaptive filtration on the quality of data transmission in a hydroacoustic channel in difficult propagation conditions. By applying these two techniques, an increase in the quality of digital data reception was expected. This quality is understood as the ability to correctly distinguish the state of individual transmitted bits.

To achieve the main purpose, a number of simulation tests were carried out, and then the obtained results were verified in real conditions. It should be emphasized that the results obtained during the tests under real conditions are consistent with the results of the simulation tests. As a result of the research carried out, it was found, inter alia, that the use of the adaptive RLS filter is justified only when the value of the total power of the replicas is sufficiently large in relation to the signal’s first replica. Moreover, even with a large number of replicas and their relatively high total power, RLS adaptive filtering ensures improvement in the quality. Using diversity combining is always justified. It should be noted that the collective reception reduces the SNR, which increases the applicability of the RLS filtration. If the total power of the replicas decreases practically at least 10 times (in relation to the first signal), the noise determines the quality. In such a case, the traditional reception should be used. In addition, diversity combining reduces inter-symbol interference, i.e., the amplitudes of the replicas (echoes) of the transmitted signal decrease approximately as many times as the number of used reception channels. This situation occurs only if signal’s first replica is subject to the coherent addition and the impulse responses for each channels are uncorellated.

In future studies, indicators enabling automatic activation of RLS adaptive filtering will be researched, and other methods of mixing signals in the receiver during diversity combining will be tested. Additionally, research has been planned to check the impact of these two techniques on the transmission quality using other modulation methods (QPSK (Quadrature Phase Shift Keying), 8PSK, 16PSK and so on) as well as other transmission techniques, such as Orthogonal Frequency-Division Multiplexing or Fast Frequency Hopping, all in difficult propagation conditions. Hardware implementation of the methods for the application to real-time processing systems will also be considered.

## Figures and Tables

**Table 1:** Results for data transmission at 4 m and 8 m distances between the hydrophones and the transmitter.

	without RLS		with RLS	
	BER	$I_{quality}$	BER	$I_{quality}$
Hydrophone at distance 4 m	0.033	4.257	0.003	7.242
Hydrophone at distance 8 m	0.040	4.148	<10^−3^	10.345
After diversity combining	0.005	7.484	<10^−4^	15.867

**Table 2:** Results for data transmission at 24 m and 28 m distances between the hydrophones and the transmitter.

	without RLS		with RLS	
	BER	$I_{quality}$	BER	$I_{quality}$
Hydrophone at distance 24 m	0.066	2.940	0.417	0.155
Hydrophone at distance 28 m	0.111	2.180	0.496	0.038
After diversity combining	0.030	3.985	0.410	0.214

## References

[1] Stojanovic M., Preisig J. (2009). Underwater acoustic communication channels: Propagation models and statistical characterization. IEEE Commun. Mag..

[2] Kochańska I. (2020). Reliable OFDM Data Transmission with Pilot Tones and Error-Correction Coding in Shallow Underwater Acoustic Channel. Appl. Sci..

[3] Czapiewska A., Luksza A., Studanski R., Zak A. (2020). Reduction of the Multipath Propagation Effect in a Hydroacoustic Channel Using Filtration in Cepstrum. Sensors.

[4] Kochańska I. (2020). Assessment of Wide-Sense Stationarity of an Underwater Acoustic Channel Based on a Pseudo-Random Binary Sequence Probe Signal. Appl. Sci..

[5] Studanski R., Zak A. (2017). Results of impulse response measurements in real conditions. J. Mar. Eng. Technol..

[6] Kochańska I., Schmidt J.H., Marszal J. (2020). Shallow Water Experiment of OFDM Underwater Acoustic Communications. Arch. Acoust..

[7] Zhou S., Wang Z.-H. (2014). OFDM for Underwater Acoustic Communications.

[8] Melodia T., Kulhandjian H., Kuo L., Demirors E., Basagni S., Conti M., Giordano S., Stojmenovic I. (2013). Advances in Underwater Acoustic Networking. Mobile Ad Hoc Networking: Cutting Edge Directions.

[9] Demirors E., Sklivanitis G., Melodia T., Batalama S.N., Pados D.A. (2015). Software-defined Underwater Acoustic Networks: Toward a High-rate Real-time Reconfigurable Modem. IEEE Commun. Mag..

[10] Tsimenidis C.C., Hinton O.R., Adams A.E., Sharif B.S. (2001). Underwater Acoustic Receiver Employing Direct-Sequence Spread Spectrum and Spatial Diversity Combining for Shallow-Water Multiaccess Networking. IEEE J. Ocean. Eng..

[11] Borowski B. (2009). Characterization of a very shallow water acoustic communication channel. Oceans.

[12] Songa H.C., Kimb J.S., Hodgkiss W.S., Kuperman W.A. (2010). High-rate multiuser communications in shallow water. J. Acoust. Soc. Am..

[13] McGee J.A., Catipovic J., Swaszek P.F. (2014). Leveraging Spatial Diversity to Mitigate Interference in Underwater Acoustic Communication Networks. J. Acoust. Soc. Am..

[14] McGee J.A. (2015). Applying Diversity to Mitigate Interference in Underwater Acoustic Communication Networks. Ph.D. Thesis.

[15] Seo B.-M., Cho H.-S. A Multipath Diversity Combining in Underwater CDMA System. Proceedings of the 11th ACM International Conference on Underwater Networks & Systems.

[16] Song H.C., Hodgkiss W.S. (2012). Diversity combining for long-range acoustic communication in deep water. J. Acoust. Soc. Am..

[17] Ishimoto R., Yoshizawa S., Tanimoto H., Saito T. Evaluation of Diversity Combining Methods for Underwater Acoustic OFDM Communication. Proceedings of the IEEE International Symposium on Intelligent Signal Processing and Communication Systems (ISPACS).

[18] Saotome R., Hai T.M., Matsuda Y., Suzuki T., Wada T. (2015). An OFDM Receiver with Frequency Domain Diversity Combined Impulsive Noise Canceller for Underwater Network. Sci. World J..

[19] Abdi A., Guo H. (2009). A new compact multichannel receiver for underwater wireless communication networks. IEEE Trans. Wirel. Commun..

[20] Brennan D.G. (1959). Linear diversity combining techniques. Proc. IRE.

[21] Moon J., Kim Y. (2003). Antenna Diversity Strengthens Wireless LANs. Commun. Syst. Des..

[22] Lindenmeier S.M., Reiter L.M., Barie D.E., Hopf J.F. Antenna Diversity for Improving the BER in Mobile Digital Radio Reception Especially in Areas with Dense Foliage. Proceedings of the International ITG Conference on Antennas.

[23] Zhang G., Dong H. (2001). Spatial diversity in multichannel processing for underwater acoustic communications. Ocean Eng..

[24] Song H.C., Hodgkiss W.S., Kuperman W.A., Stevenson M., Akal T. (2006). Improvement of Time-Reversal Communications Using Adaptive Channel Equalizers. IEEE J. Ocean. Eng..

[25] Pelekanakis K., Chitre M. Comparison of sparse adaptive filters for underwater acoustic channel equalization/Estimation. Proceedings of the IEEE International Conference on Communication Systems.

[26] Tao J., Wu Y., Wang X., Luo X. Comparison of Sparsity-Aware LMS Adaptive Equalization for Underwater Acoustic Communications. Proceedings of the OCEANS-MTS/IEEE Kobe Techno-Oceans (OTO).

[27] Tong F., Benson B., Li Y., Kastner R. Channel Equalization Based on Data Reuse LMS Algorithm for Shallow Water Acoustic Communication. Proceedings of the IEEE International Conference on Sensor Networks, Ubiquitous, and Trustworthy Computing.

[28] Xiao Y., Yin F. (2014). Blind equalization based on RLS algorithm using adaptive forgetting factor for underwater acoustic channel. China Ocean Eng..

[29] Pelekanakis K., Chitre M. (2015). Robust Equalization of Mobile Underwater Acoustic Channels. IEEE J. Ocean. Eng..

[30] Patra J.C., Pal R.N. (1995). A functional link artificial neural network for adaptive channel equalization. Signal Process..

[31] Kechriotis G., Zervas E., Manolakos E.S. (1994). Using recurrent neural networks for adaptive communication channel equalization. IEEE Trans. Neural Netw..

[32] Goel S., Abawajy J.H., Kim T.-H. (2010). Performance Analysis of Receive Diversity in Wireless Sensor Networks over GBSBE Models. Sensors.

[33] Compare RLS and LMS Adaptive Filter Algorithms-MathWorks Help Center. https://www.mathworks.com/help/dsp/ug/compare-rls-and-lms-adaptive-filter-algorithms.html.

[34] Gregg W.D. (1977). Analog and Digital Communication.

